# Bedside Endoscopic Retrograde Cholangiopancreatography Using Portable X-Ray in Acute Severe Cholangitis

**DOI:** 10.1155/2018/8763671

**Published:** 2018-02-28

**Authors:** Rushikesh Shah, Emad Qayed

**Affiliations:** Department of Medicine, Division of Digestive Diseases, Emory University School of Medicine, Atlanta, GA, USA

## Abstract

Patients with acute cholangitis require emergent biliary decompression. Those who are hemodynamically unstable on vasopressor support and mechanical ventilation are too critically ill to move outside of the intensive care unit. This prohibits performing Endoscopic Retrograde Cholangiopancreatography (ERCP) in the endoscopy unit. Fluoroscopic guidance is required to confirm deep biliary cannulation during ERCP. There are a few reported cases of bedside ERCP using portable C-arm fluoroscopy unit or ultrasound guided cannulation. We present a unique case of life-saving emergent bedside ERCP in a severely ill patient with cholangitis and septic shock, using simple portable X-ray to confirm biliary cannulation.

## 1. Introduction

Patients with severe cholangitis associated with septic shock, organ failure, and no response to antibiotics (Tokyo classification grade 3) have high mortality and morbidity. These patients require emergent biliary decompression with Endoscopic Retrograde Cholangiopancreatography (ERCP) [1]. However, patients who are hemodynamically unstable on vasopressor support and mechanical ventilation are too critically ill to move outside of the intensive care unit (ICU). There are limited options for radiologic guidance to confirm cannulation during ERCP in the ICU setting. We present a unique case in which life-saving bedside ERCP in the ICU was successful, using one abdominal radiograph obtained with a portable X-ray machine.

## 2. Case Report

An octogenarian male presented to the emergency room with worsening generalized abdominal pain, nausea, and fatigue of three-day duration. He had a history of choledocholithiasis and distal common bile duct stricture, for which he underwent ERCP with sphincterotomy, biliary dilation, and biliary stent placement two years prior to presentation. The patient did not follow up for repeat ERCP and did not undergo cholecystectomy. On presentation, his vital signs were as follows: BP 110/62, HR 101/min, T 99 F, and RR 12/min. Physical examination revealed a soft abdomen without tenderness. Laboratory testing showed total bilirubin of 2.6 mg/dl (direct of 1.1 mg/dl), AST 75 U/L, ALT 30 U/L, and creatinine of 0.7 mg/dL. Computed tomography scan showed dilated intra- and extrahepatic bile ducts with biliary stent in the common bile duct (CBD) terminating at the level of ampulla. The gallbladder was distended with a large dependent gallstone, without gallbladder wall thickening. The patient was admitted to the floor with plans to perform ERCP the next morning. Overnight, he developed delirium and became unstable. His repeat vital signs showed BP 80/50, HR 120/min, T 102 F, and RR 35/min. He was transferred to the ICU with the diagnosis of cholangitis and septic shock. Repeat metabolic panel showed total bilirubin of 5.4 mg/dl (direct 2.6), AST 206 U/L, ALT 180 U/L, and creatinine 1.5 mg/dl. He quickly deteriorated and developed cardiac arrest, with pulseless electrical activity requiring cardiopulmonary resuscitation for few minutes, with return of spontaneous circulation. At this point he was on intravenous fluids, three vasopressors, broad-spectrum antibiotics (piperacillin and tazobactam), and mechanical ventilation. Due to his critical illness, it was decided to perform a bedside ERCP for emergent decompression.

**Figure 1:**
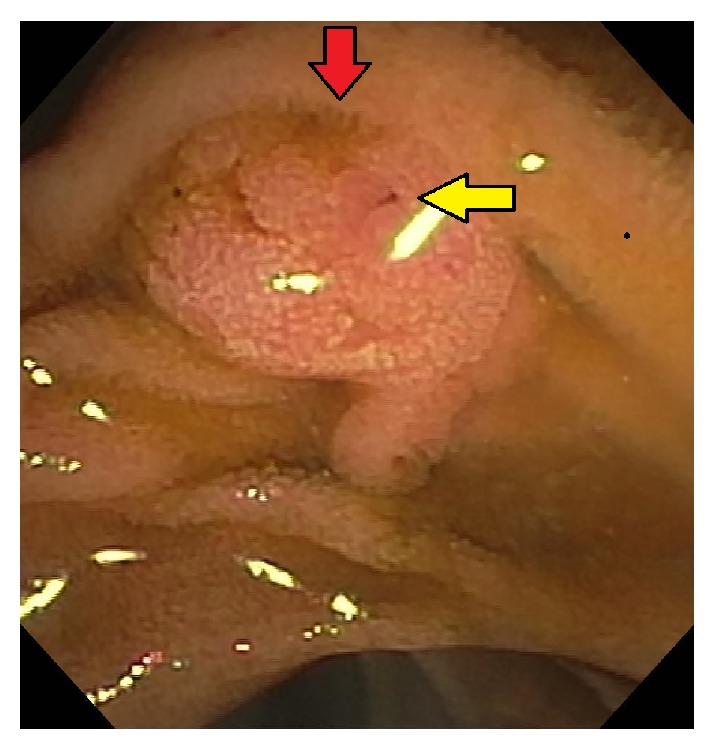
Ampulla prior to cannulation. The biliary orifice (red arrow) and pancreatic orifice (yellow arrow) are seen.

**Figure 2:**
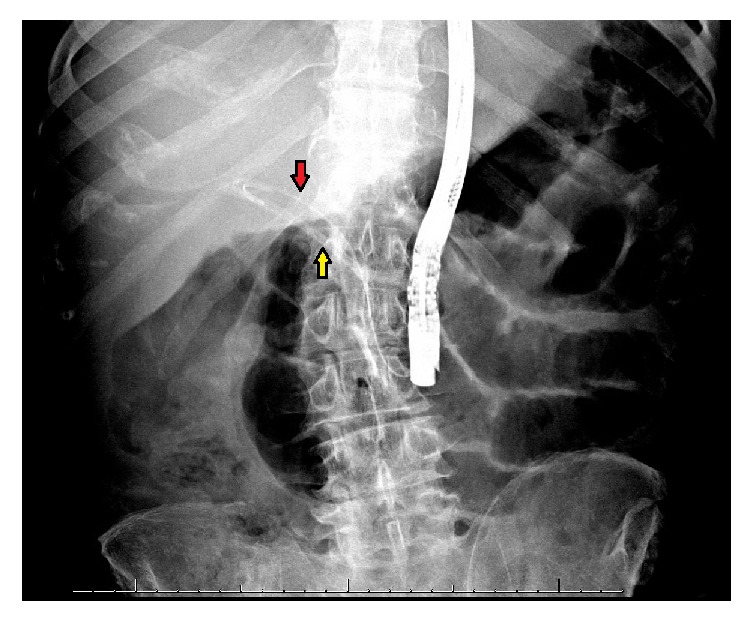
Abdominal radiograph obtained during bedside ERCP showing sphincterotome inside the common bile duct (red arrow). The migrated stent (yellow arrow) is seen with the distal tip inside the bile duct.

**Figure 3:**
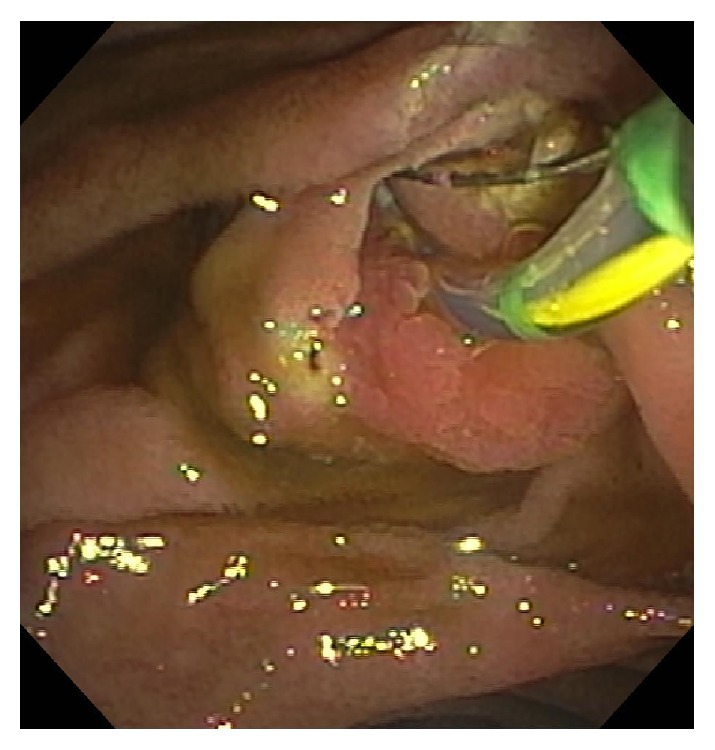
Extension sphincterotomy.

**Figure 4:**
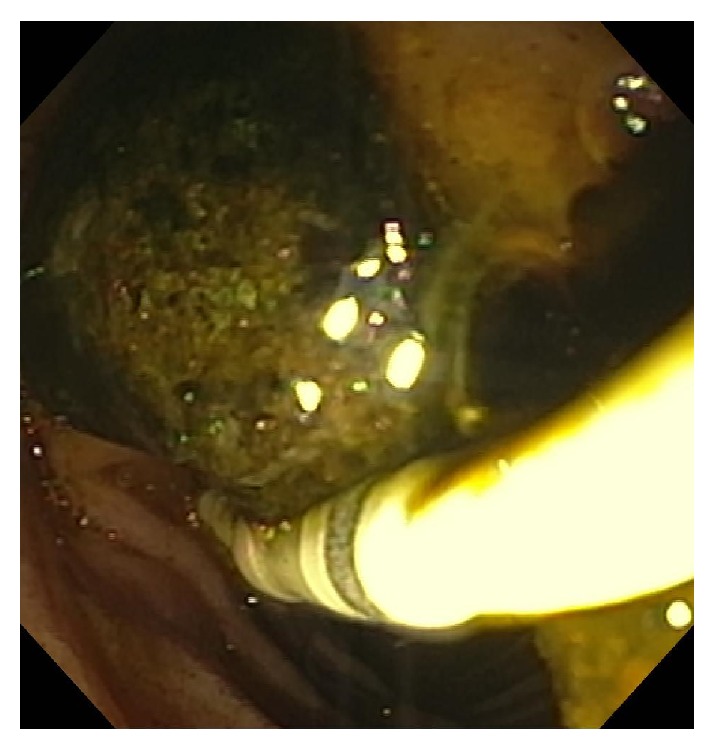
Stone extraction.

**Figure 5:**
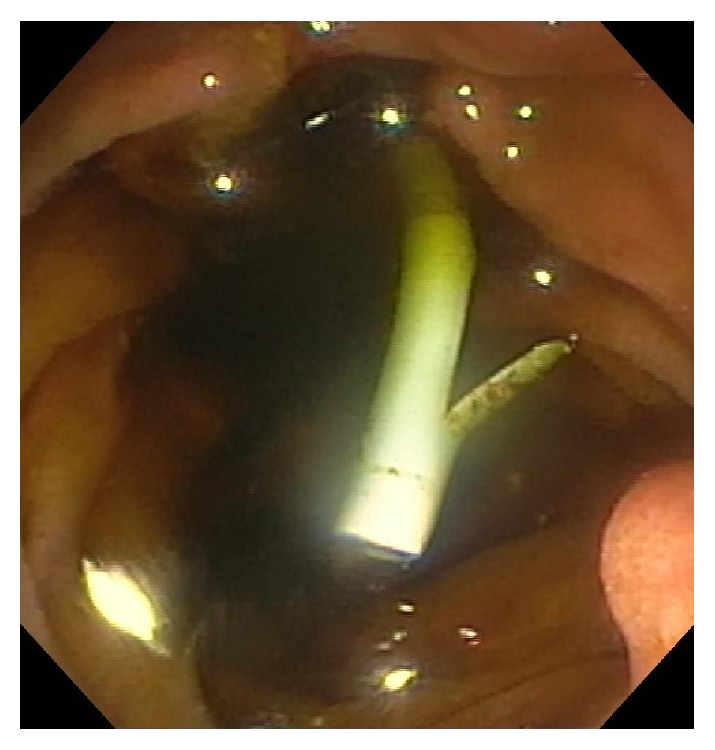
Common bile duct stent placement.

**Figure 6:**
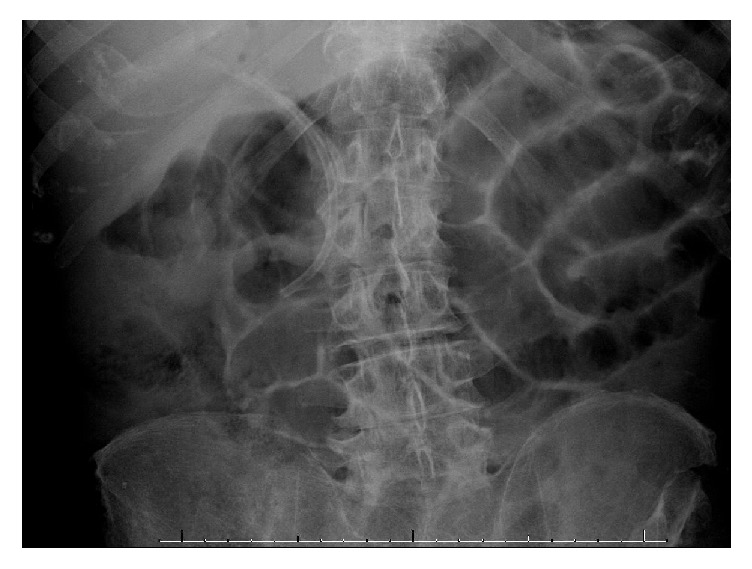
Abdominal radiograph showing appropriate position of the new plastic stent. The old, migrated stent was left inside the bile duct.

The patient remained in his ICU bed in the supine position. The endoscopy cart and electrosurgical generator were set up at his bedside in the usual fashion. Propofol infusion was used for sedation. The portable X-ray technician was informed that an immediate “STAT” X-ray would be needed during the procedure. During ERCP, the biliary stent was not visible through the ampulla, suggesting that it had migrated proximally into the bile duct. The biliary orifice was identified in the superior aspect of the ampulla (Figure 1). The wire was easily inserted into the biliary orifice, followed by insertion of the sphincterotome. The patient was repositioned slightly to place the X-ray cassette behind his back. The X-ray was obtained and uploaded to the electronic medical record immediately. The position of the sphincterotome was confirmed in the CBD, and the stent was seen in the proximal bile duct (Figure 2). Contrast injection was not required. An extension sphincterotomy was performed (Figure 3). Blind balloon sweeps were performed using a 12 mm balloon-tipped catheter, and several green stones were extracted (Figure 4). The previous stent could not be seen or retrieved. A 10 Fr, 9 cm plastic stent was inserted over the wire into the CBD without X-ray guidance (Figure 5). Copious bile flow through the stent was observed. The scope was withdrawn at this time, with a total procedure time of 34 minutes. Appropriate stent placement was confirmed with a repeat abdominal X-ray (Figure 6). The patient improved significantly over the next few hours, and all vasopressors were weaned off. Blood cultures grew* Escherichia coli*. He was extubated the next morning and transferred to the floor one day later. A repeat inpatient ERCP was performed one week later in the endoscopy unit under general anesthesia. Both stents were retrieved, and multiple bile duct stones were extracted. The patient was discharged home the next day in good condition.

## 3. Discussion

Patients with severe cholangitis require emergent decompression. While the appropriate timing of the endoscopy is not entirely clear, sepsis guidelines suggest source control within 12 hours to improve survival in patients with septic shock [2]. These patients are usually on multiple infusions, are receiving mechanical ventilation, and are too sick to be transported outside of the ICU. Therefore, emergent bedside ERCP should be considered in this setting. Previous case series described bedside ERCP using fluoroscopic C-arm [3]. However, fluoroscopic equipment may not be available, are difficult to use in the ICU, and require a fluoroscopically compatible bed. A regular ICU bed requires the operator to angle the C-arm to avoid the opacities in the bed frame.

Transabdominal and intraductal ultrasound have been used to confirm CBD cannulation during bedside ERCP [4]. The use of these techniques depends on availability of equipment and local expertise. Previous reports described ERCP without fluoroscopic guidance, in which biliary cannulation is confirmed with aspiration of bile. However, this method does not guarantee deep CBD cannulation and risks the accidental stent placement in the cystic duct, leading to failed biliary decompression.

To our knowledge, this is the first reported case of bedside ERCP using a simple portable X-ray method to confirm cannulation and proceed with therapeutic intervention to treat acute severe cholangitis. Regardless of the method used to confirm cannulation of the CBD, bedside ERCP should be considered in critically ill patients with cholangitis, in whom the suspected obstruction is in the extrahepatic bile ducts. Prior sphincterotomy and/or stent placement renders cannulation straightforward. This should encourage the endoscopist to attempt bedside ERCP and not move a critically ill patient to the operating room or GI unit. In this case, using a simple portable X-ray confirmed successful biliary cannulation and allowed for successful stent placement. Bedside ERCP can be life-saving, and could reduce mortality and morbidity in patients with severe cholangitis.
